# Mononeuritis Multiplex as a Diagnostic Challenge in Eosinophilic Granulomatosis With Polyangiitis: Implications of Early Versus Delayed Recognition

**DOI:** 10.7759/cureus.93171

**Published:** 2025-09-25

**Authors:** Farhaan Ahmed, Hamna Nazeer, Salamat Ullah, Rawaha Ahmad, Nichola Pugh

**Affiliations:** 1 Internal Medicine, Northampton General Hospital NHS Trust, Northampton, GBR; 2 Acute Medicine, Northampton General Hospital NHS Trust, Northampton, GBR; 3 Rheumatology, Northampton General Hospital NHS Trust, Northampton, GBR; 4 Pulmonology, Northampton General Hospital NHS Trust, Northampton, GBR

**Keywords:** anti-il-5 therapy, asthma, corticosteroids, eosinophilic granulomatosis with polyangiitis (egpa), mononeuritis multiplex

## Abstract

A 63-year-old female patient presented to the acute medical unit with what appeared to be a case of progressive mobility decline, a common and often nonspecific presentation in acute medicine. She described upper and lower limb weakness and numbness affecting her ability to walk. Originally, her upper limb numbness was treated as ulnar nerve compression in the community. She also had multiple episodes of shortness of breath throughout the year, which were initially managed in the community as suspected asthma exacerbations; however, her symptoms were non-specific. Routine investigations revealed severe eosinophilia (13.37 × 10⁹/L), anaemia, and leucocytosis. Neurophysiology confirmed mononeuritis multiplex, and further autoimmune testing revealed positive anti-neutrophil cytoplasmic antibodies (P-ANCA) and elevated myeloperoxidase (MPO) antibodies. This was ultimately diagnosed as eosinophilic granulomatosis with polyangiitis (EGPA), a rare presentation that challenged initial diagnostic assumptions.

This case is an example of how not all presentations of EGPA are straightforward and how it might be missed in an acute medical setting. Through this case report, we wanted to emphasise the subtle and often vague nature of mononeuritis multiplex in EGPA.

We would also like to highlight the implications of a delayed diagnosis on patients with EGPA and how this affects the long-term health of patients. Lastly, we would like to discuss newer treatments with anti-interleukin-5 (IL-5) target drugs, which are used to reduce relapse rates in the disease.

## Introduction

Eosinophilic granulomatosis with polyangiitis (EGPA), previously known as Churg-Strauss syndrome, is a rare form of anti-neutrophil cytoplasmic antibodies (ANCA)-associated vasculitis that includes small- to medium-vessel vasculitis. Briefly, this means that ANCAs are involved in an autoimmune attack and eosinophil-driven inflammation of vessels, which leads to vessel and tissue damage [1]. Described for the first time in 1951 by Churg and Strauss, EGPA is part of the spectrum of ANCA-associated vasculitides together with granulomatosis with polyangiitis (GPA) and microscopic polyangiitis (MPA) [2]. The 2022 American College of Rheumatology/European Alliance of Associations for Rheumatology (ACR/EULAR) EGPA criteria include eosinophilia, obstructive disease (more commonly asthma), extravascular eosinophilic-predominant involvement, nasal polyps, mononeuritis multiplex/motor neuropathy, haematuria and ANCA positivity [3].

Eosinophils play a key role in the immunopathogenesis of EGPA; they mediate tissue damage, which can be supported by the effectiveness of IL-5-blocking drugs. Innate lymphoid cells encourage eosinophil recruitment, T cells drive inflammation, and B cells contribute by producing ANCA and IgG4 [4].

EGPA typically has three phases: prodromal phase marked by atopic disease such as allergic rhinitis and asthma; eosinophilic phase characterized by blood and tissue eosinophilia (accumulation of increased number of eosinophils within tissue) involving the lung and gastrointestinal tract; and the vasculitic phase which is depicted by necrotizing vasculitis (autoimmune mediated cell death of vessel walls) often affecting skin, nerves, kidneys and heart [5].

Neurological involvement is a common presentation in EGPA, affecting approximately 60-70% of the patients [6]. Mononeuritis multiplex, resulting from the necrotising vasculitis of the vasa nervorum, leads to asymmetric sensorimotor deficits commonly affecting the peroneal and ulnar nerve [7]. Patients may exhibit paraesthesia, wrist drop, or foot drop, while peripheral polyneuropathy and cranial nerve involvement are less common [5]. Since prompt immunosuppressive treatment can prevent irreversible nerve damage, early detection of neurological symptoms is crucial. Investigations such as electromyography and nerve conduction studies are frequently useful in the diagnostic process [8].

## Case presentation

A 63-year-old female patient was admitted to the emergency department with a two-month progressive mobility decline. She reported worsening lower limb weakness and numbness. Originally, this started in the upper limb but then progressed to the lower limb, ultimately affecting her ability to walk. Notably, her medical history revealed that one year prior to this, she presented to her general practitioner with multiple episodes of shortness of breath, which were presumed to be infective exacerbations of asthma. She was diagnosed with asthma around six years ago. This was clinically diagnosed in the community by her general practitioner. Over the course of a year, multiple steroid and antibiotic regimens were prescribed. Routine blood tests revealed a raised white cell count, reduced haemoglobin (Hb) level and a high eosinophil count (Table 1).

**Table 1:** Blood test result on presentation ANCA: anti-neutrophil cytoplasmic antibodies; MPO: myeloperoxidase; PR3: proteinase 3

Test	Result	Reference range	Interpretation
C-reactive protein (CRP)	48 mg/L	< 5 mg/L	Elevated – active inflammation
Haemoglobin (Hb)	89 g/L	115–160 g/L (female)	Low – normocytic anaemia
White cell count (WCC)	21.6 ×10⁹/L	4.0–11.0 × 10⁹/L	Elevated – leucocytosis
Eosinophils	13.37 ×10⁹/L	0.0–0.4 × 10⁹/L	Severe eosinophilia
Platelets	640 ×10⁹/L	150–400 × 10⁹/L	Elevated – thrombocytosis
Beta-2 microglobulin	3.67 mg/L	0.7–1.8 mg/L	Elevated – possible immune activation
ANCA	Positive (p-ANCA)	Negative	Positive – perinuclear ANCA pattern
MPO antibodies	416 U/mL	< 20 U/mL	Significantly elevated – MPO-ANCA
PR3 antibodies	Negative	< 20 U/mL	Within normal range

Concurrently, the patient began to experience loss of sensation and power in both of her hands. This initially started around two months ago and involved the fourth and fifth fingers of the right hand. This was another issue investigated by the GP and treated as an ulnar nerve compression. Physiotherapy was recommended, and the patient was discharged.

Upon admission to the hospital, the patient similarly complained of progressive loss of sensation in both hands. This started on the right hand, including the 4th and 5th fingers and progressed to the opposite side with the same distribution. The sensory loss progressed to involve the lateral aspect of the calves. She also reported generalised weakness in her upper and lower limbs. Neurological examination revealed a bilateral weakness and altered sensation of the medial two fingers in the hands, with a positive Froment's sign. There was a reduced patchy sensation distally over fingers, noted with the right thumb being prominently affected. Sensation was also affected in the toes, with a patchy loss across the dorsal planes. Ankle jerks were also diminished. Muscle strength was reduced to 3/5 in the upper limbs and 4/5 in the lower limbs, with significantly impaired hand grip strength. The clinical findings were consistent with mononeuritis multiplex, and the patient met the 2022 ACR/EULAR criteria for eosinophilic granulomatosis with polyangiitis [3]. Given the recent diagnosis of asthma that was difficult to manage, the non-specific neurological symptoms, and the raised eosinophilia, a vasculitis screen was ordered to rule out vasculitis.

Investigations

**Figure 1:**
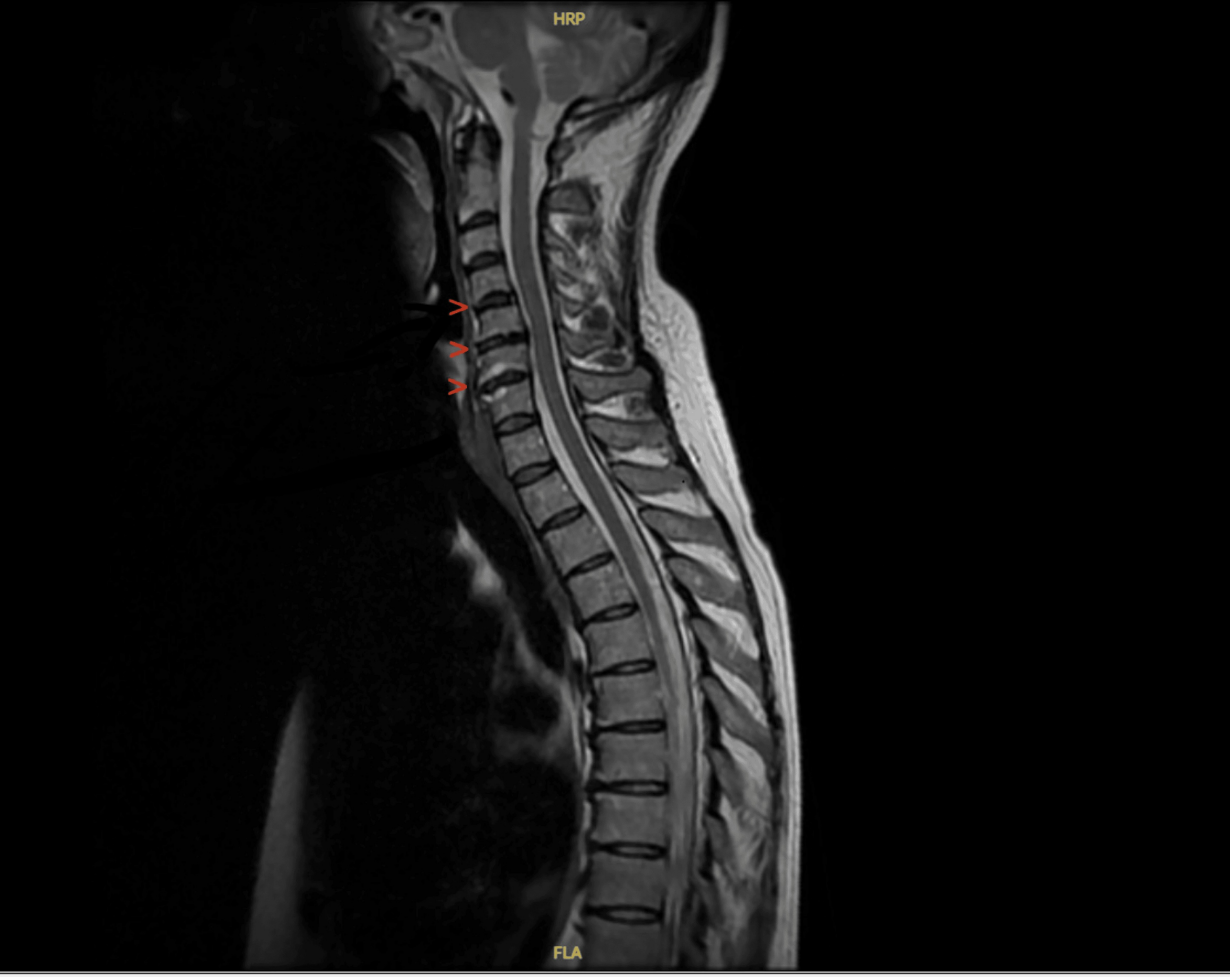
MRI whole spine showing degenerative changes between C5-C6 and C6-C7

Investigations were performed to support the diagnosis. Firstly, an MRI scan was performed of the spine. There was no acute abnormality identified, but there were some degenerative changes of discs in the cervical region (C5-C7) and in the lumbar spine (Figure 1).

**Figure 2:**
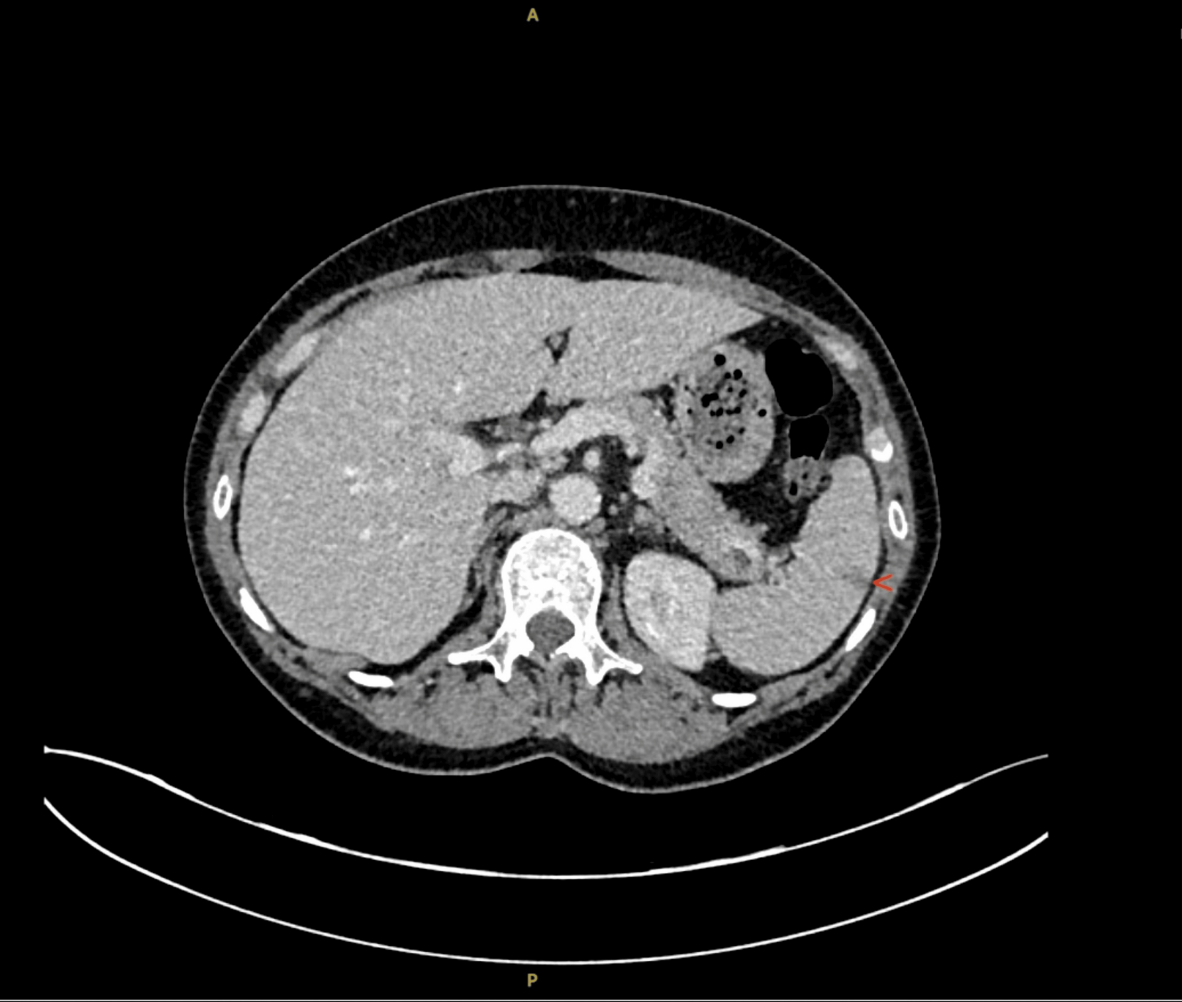
CT abdomen-pelvis showing linear peripheral hypodensity of spleen

There was a bilateral lateral recess narrowing in the spine and possible impingement of the L5 nerve root. Nerve conduction studies showed absent sensory responses in the right median, bilateral ulnar and left sural nerves. There were also reduced motor amplitudes in both limbs, keeping with rapidly progressive mononeuritis multiplex. A CT abdomen and pelvis was performed, which showed a linear hypodensity suggestive of a splenic infarct and mucous plugging in the right lung with some left lung linear atelectasis (Figure 2).

Treatment 

The initial mainstay of treatment for EGPA is with glucocorticoids. While glucocorticoids are effective in the initial stages of the disease, relapse is common following tapering or withdrawal. As a result, many patients stay on corticosteroid therapy long-term, exposing them to significant side effects of steroids. Moreover, glucocorticoids are rarely sufficient as a monotherapy and are typically used as induction therapy in the disease. Induction is usually achieved with glucocorticoids such as prednisolone, while maintenance is provided with immunosuppressants such as cyclophosphamide or rituximab [9]. In this case, the patient was started on Intravenous methylprednisolone for three days, followed by oral prednisolone and is currently on a tapering regimen. She had received six doses of intravenous cyclophosphamide.

Given the severity of her asthma and neurological symptoms, the patient was referred to a multidisciplinary meeting to consider anti-IL-5 therapy. Owing to the significant disability caused by EGPA, including loss of independence and inability to drive, she was also referred to our tertiary centre. As per regional guidelines, she was referred to the eosinophilia team in the tertiary centre for approval of the anti-IL-5 therapy. Interleukin-5 is a key mediator for eosinophils; it is responsible for stimulating proliferation, maturation and differentiation of eosinophils. As eosinophils play a central role in the pathogenesis of EGPA, anti-IL-5 therapies can specifically target the molecules for anti-eosinophilic treatments [10]. Anti-IL-5 therapies include the use of mepolizumab and benralizumab. They have emerged as effective options for patients suffering from relapsing or refractory EGPA. Although they are not routinely used in acute presentations, they are most effective for maintenance therapy. After administration, both drugs have been shown to reduce eosinophil counts within three months, lead to significant improvements in respiratory symptoms (including increased FEV1), and allow for a marked reduction in oral corticosteroid doses of more than 50% [11].

A systematic review demonstrated that mepolizumab has notably reduced relapse rates and corticosteroid dependence. In a randomised controlled trial, patients who were treated with mepolizumab alongside prednisolone experienced a 50% reduction in relapse rate, compared to those treated with prednisolone alone. Furthermore, there were reduced rates of remission in both asthmatic and vasculitic symptoms [11]. Benralizumab has shown similar efficacy, with studies reporting corticosteroid dose reductions from a median of 15 mg to 2 mg daily and a reduction in annual exacerbations from five to fewer than two episodes [12].

## Discussion

While poor mobility is a common presentation that is seen in an acute medical setting, it does not inherently lead to a definite diagnosis. It can be attributed to many different diseases, and this case portrayed the crucial role of a thorough history and physical examination. Rather than adopting a system-based approach, the physical exam allowed us to recognise vital neurological signs, which aided our diagnosis. Mononeuritis multiplex is seen in more than 60% of patients in EGPA [13]. Our patient presented with classical symptoms, and as we linked this to the patient's history, it aided us in the diagnosis.

While EGPA can present with a variety of presentations, neurology is not always the initial complaint. While peripheral neuropathies are found within EGPA, it is often misdiagnosed, as in this case, where it was initially said to be ulnar nerve compression. Early recognition and a thought of vasculitis could have significantly reduced complications in the patient [1]. In this case, vasculitic damage caused significant muscle and sensory loss, affecting the patient's mobility, ability to walk and to drive. The disability is likely to leave her with a long-term deficit despite the current management plan.

Furthermore, this highlights the importance of early recognition and investigation of vasculitis, particularly in patients with atypical or ranged symptoms. Studies have shown that, with a timely diagnosis and treatment, there is a favourable prognosis of a five-year survival rate of 90% [1]. A thorough clinical history and physical exam, alongside timely ordering of vasculitic screens, could have enabled an earlier diagnosis and potentially reduced the extent of irreversible damage. Although EGPA is not a frequent diagnosis, it should be considered in patients who have suggestive clinical features, with more focus when routine treatments for neurological and respiratory conditions have not helped.

In this case, the patient's eosinophilia was initially overlooked, as it was attributed to a presumed history of recurrent asthma exacerbations. This provided a plausible explanation at the time. The patient's background of asthma, alongside new systemic features, raised further suspicion of a vasculitis process. While eosinophilia can be seen in other conditions such as eosinophilic asthma, certain malignancies and parasitic infections [14], the presence of marked eosinophilia with systemic involvement should always warrant an evaluation for vasculitis. A broad different diagnosis was considered for this patient, including malignancy, infections and autoimmune conditions, which eventually guided us towards vasculitis.

Exacerbation of asthma and other allergic symptoms are common presentations among the general population; we need to keep a high index of suspicion to consider EGPA when there is a poor response to standard treatment. In practice, on the busy acute take, the priority is to manage acute presentation due to time and demand limitations. For example, this patient presented with reduced mobility due to sensory symptoms, and an MRI of the spine was done to rule out cord compression and a CT chest to rule out malignancy or other inflammatory causes. Only once these acute considerations were addressed did the possibility of vasculitis come to the forefront, prompting nerve conduction studies and autoimmune screening. However, these specialised investigations take additional time to complete, further contributing to diagnostic delay in these conditions.

The multi-disciplinary team (MDT) played a critical role in further management. The patient was referred to Rheumatology due to suspicion of vasculitis, as well as to neurology for ongoing neurological symptoms, for further input and nerve conduction studies. The collaboration between all teams was essential in guiding the diagnostic process and management plan. The Neurology team performed nerve conduction studies, which confirmed the presence of mononeuritis multiplex, a key manifestation of EGPA. The Rheumatology team ordered and interpreted relevant autoimmune serology, including ANCA antibodies. They also helped by correlating the serology findings with the clinical picture, therefore reinforcing diagnostic certainty. While tissue diagnosis remains the gold standard for vasculitis [15], in this case, it was not requested initially as the patient met the ACR/EULAR classification criteria for EGPA [3]. The diagnosis was clinically sound, supported by electromyography (EMG) findings and serology. The MDT input led to early induction therapy after diagnosis.

This case also emphasises the role of anti-IL-5 in maintenance treatment of the disease. Mepolizumab and benralizumab have shown promise in reducing relapse rates and corticosteroid dependence. The treatment should be considered in patients who suffer from relapsing disease and disease which is inadequately controlled by glucocorticoids alone [12].

## Conclusions

Although the patient had symptoms, such a asthma exacerbations and mobility decline, they were not linked, resulting in adverse effects on the patient in terms of her prognosis. In a busy setting such as the acute medical unit, physicians should have a higher index of suspicion for vasculitis conditions in patients with unexplained multisystem features, especially when regular treatments have failed. Additionally, this case supports the use of anti-IL-5 medications in patients with relapsing EGPA. 

Initially, the presentation was challenging, as the symptoms were not specific, such as numbness and weakness, and didn’t seem related. On pattern recognition, we reached a final diagnosis. This case highlights the importance of detailed clinical history with pattern recognition and the importance of good physical examination. This also highlights that we should always think outside the box when there is a diagnostic dilemma.
